# Ribavirin for Chronic Hepatitis Prevention among Patients with Hematologic Malignancies

**DOI:** 10.3201/eid2108.150199

**Published:** 2015-08

**Authors:** Suzanne Tavitian, Jean-Marie Peron, Françoise Huguet, Nassim Kamar, Florence Abravanel, Odile Beyne-Rauzy, Lucie Oberic, Stanislas Faguer, Laurent Alric, Murielle Roussel, Clément Gaudin, Loïc Ysebaert, Anne Huynh, Christian Recher

**Affiliations:** Université Paul Sabatier, Toulouse (S. Tavitian, J.-M. Peron, O. Beyne-Rauzy, L. Alric, C. Gaudin, C. Recher);; Institut Universitaire du Cancer, Toulouse, France (S. Tavitian, F. Huguet, O. Beyne-Rauzy, L. Oberic, M. Roussel, L. Ysebaert, A. Huynh, C. Recher);; Centre Hospitalier Universitaire, Toulouse, France (J.-.M Peron, N. Kamar, F. Abravanel, S. Faguer, L. Alric, C. Gaudin)

**Keywords:** Hepatitis E, hematology, malignancy, chronic hepatitis, cirrhosis, ribavirin, viruses

## Abstract

Findings among a cohort of 26 patients who had hematologic malignancies and hepatitis E virus (HEV) infection support that HEV can induce chronic hepatitis. However, a 3-month course of ribavirin can induce a rapid viral clearance, reducing the risk for chronic hepatitis and enabling continuation of cytotoxic treatments for underlying malignancies.

Hepatitis E virus (HEV) is a nonenveloped RNA virus transmitted by consumption of contaminated water, undercooked infected pork or wild boar, deer, or rabbit meats (*1*), or by blood transfusion or solid organ transplant (SOT) (*2*). In addition to acute hepatitis, HEV causes chronic hepatitis and cirrhosis in immunocompromised patients (*3*,*4*). Knowledge of hepatitis E in patients with hematologic cancers is mostly derived from case reports or small series (*5*–*10*). We previously described 6 patients who had various hematological malignancies and HEV infection and reported chronic hepatitis in 3 of them (*6*). Management of HEV infection in the context of allogeneic stem cell transplantation (SCT) has been a matter of controversy (*5*,*6*). Ribavirin is the treatment of choice in patients infected during SOT (*11*). In hematologic patients infected with HEV, data concerning efficacy and safety of ribavirin are scarce (*12*).

## The Study

During 2003–2009, patients at Institut Universitaire du Cancer, Toulouse, were randomly tested for HEV infection; patients who had elevated liver enzymes were routinely assessed for the virus. Patients were generally counseled to avoid undercooked foods or products that increased risk for HEV transmission according to guidelines from the French Ministry of Health. All patients with a diagnosis of HEV infection were counseled. HEV RNA was detected and quantified by PCR testing, and HEV IgM and HEV IgG were assessed with commercially available kits: the EIAgen HEV IgG and IgM kits (Adaltis, Casalecchio Di Reno, Italy) before 2012, and the Wantai HEV IgG and IgM enzyme-linked immunosorbent assay (Beijing Wantai Biologic Pharmacy Enterprise Co., Ltd, Beijing, China) since 2012. Diagnosis of hepatitis E required liver enzyme abnormalities and detectable HEV RNA in serum or fecal samples. Chronic HEV infection was defined by HEV viremia lasting >3 months (*1*). 

Ribavirin monotherapy was proposed for infected immunocompromised patients beginning in May 2010, after publication of preliminary data reporting its efficacy in such patients (*12*). Assessment for administering ribavirin treatment was made on a case-by-case basis according to clinical and therapeutic context. Although there was no formal protocol, the general consensus was to consider ribavirin with the intent to complete treatment for the underlying disease rather than biologic criteria such as alanine aminotransferase levels or HEV viral loads. This study was approved by our institutional review board.

During 2003–2014, we identified 26 patients whose laboratory test results showed HEV replication. Clinical characteristics and treatments of patients with hematologic malignancies before or within the 6 months after the diagnosis of hepatitis E are summarized in Table 1 and in the Technical Appendix Table.

**Table 1:** Clinical characteristics of 26 patients subsequently diagnosed with hepatitis E and hematologic malignancy, University Hospital of Toulouse, France, 2003*

Characteristic	Value	Ribavirin		p value
		Yes, n = 12	No, n = 14	
Sex, M/F	17/9 (65/35)	7/5	10/4	0.40
Age, median y (range)†	59 (21–86)	55 (21–86)	63 (33–84)	0.55
Hematological malignancy				
Acute leukemia	9 (34.6)	3 (25)	6 (42.8%)	NA
Indolent NHL‡	8 (26.9)	4 (33.5)	4 (28.6%)	NA
Aggressive NHL§	3 (15.3)	2 (1)	1 (7.2%)	NA
Multiple myeloma	3 (11.6)	1 (8.5)	2 (14.3%)	NA
Others¶	3 (11.6)	2 (17)	1 (7.2%)	NA
SCT before or concomitant to hepatitis E				
Allogenetic SCT	1/3	0/2	1/1	NA
Hepatitis E				
Serology, n = 23				
IgM+ IgG+	6 (26.1)	2 (16.7)	4 (28.6%)	NA
IgM– IgG+	3 (13)	1 (8.4)	2 (14.3%)	NA
IgM+ IgG–	7 (30.5)	3 (25)	4 (28.6%)	NA
IgM– IgG–	7 (30.5)	4 (33.5)	3 (21.5%)	NA
At onset				
AST, IU/L (range)	150 (17–2,309)	92 (17–1,688)	179 (20–2,309)	0.30
ALT, IU/L (range)	293 (24–4,273)	297 (24–2,189)	278 (47–4,273)	0.74
γGT, IU/L (range)	202 (18–1,665)	220 (50–1,665)	181 (18–492)	0.70
Bilirubin, μmol/L (range)	13 (6.3–107)	16 (6.3–107)	13 (7.7–88)	0.43
At month 6				
AST, IU/L (range)	51 (14–1,127)	19.5 (14–325)	86 (38–1,127)	0.03
ALT, IU/L (range)	50 (10–1,523)	14 (10–446)	133 (39–1,523)	0.01
γGT, IU/L (range)	101 (14–1,375)	28 (14–1,375)	135 (34–671)	0.07
Bilirubin, μmol/L (range)	18 (6.1–2,61)	18 (7.4–261)	12 (6.1–37)	0.41

*Values are no. (%) patients except as indicated. NA, not applicable; NHL, non-Hodgkin lymphoma; SCT, stem cell transplant; AST, aspartate aminotransferase; ALT,alanine aminotransferase; γGT, gamma-glutamyl transferase. †Age at diagnosis of HEV infection. ‡Indolent B cell lymphoma: follicular lymphoma (n = 2). §Aggressive non-Hodgkin lymphoma: Burkitt-like B cell lymphoma (n = 1), anaplastic T-cell lymphoma (n = 1), Diffuse large B cell lymphoma (n = 2). ¶Others: myeloproliferative neoplasm (n = 1); granulocytic sarcoma (n = 1); myeloid variant of hypereosinophilia with FIP1L1-PDGFRα rearrangement. p value refers to comparison between patients who received ribavirin and patients who did not. Data from 6 patients of this cohort were previously reported in a preliminary study (*6*).

The diagnosis of HEV infection occurred a median of 10 (range 0–227) months after identification of the hematologic disorder. No persons in the study had traveled outside France during the year before hepatitis was diagnosed. Primary signs and symptoms were fever, diffuse or abdominal pain, vomiting, or asthenia; 16 patients were asymptomatic. Liver enzyme levels ranged from 1.5–100× the upper limit of normal (Figure). No patient had fulminant hepatitis. HEV RNA was detectable in the serum of all patients and in the fecal samples of 88% of patients (HEV strain genotype 3f in 73%, 3c in 23%, and 4b in 4%). Of 23 tested patients, 9 (36%) had HEV IgG and 13 (61%) had HEV IgM. This finding emphasizes the need to rely on HEV RNA detection rather than serologic results for HEV diagnosis in immunocompromised patients. HEV was diagnosed concomitantly to the onset (n = 2) or at relapse (n = 2) of the hematologic malignancy, which raises the hypothesis of an association between HEV infection and carcinogenesis (*13*).

**Figure:**
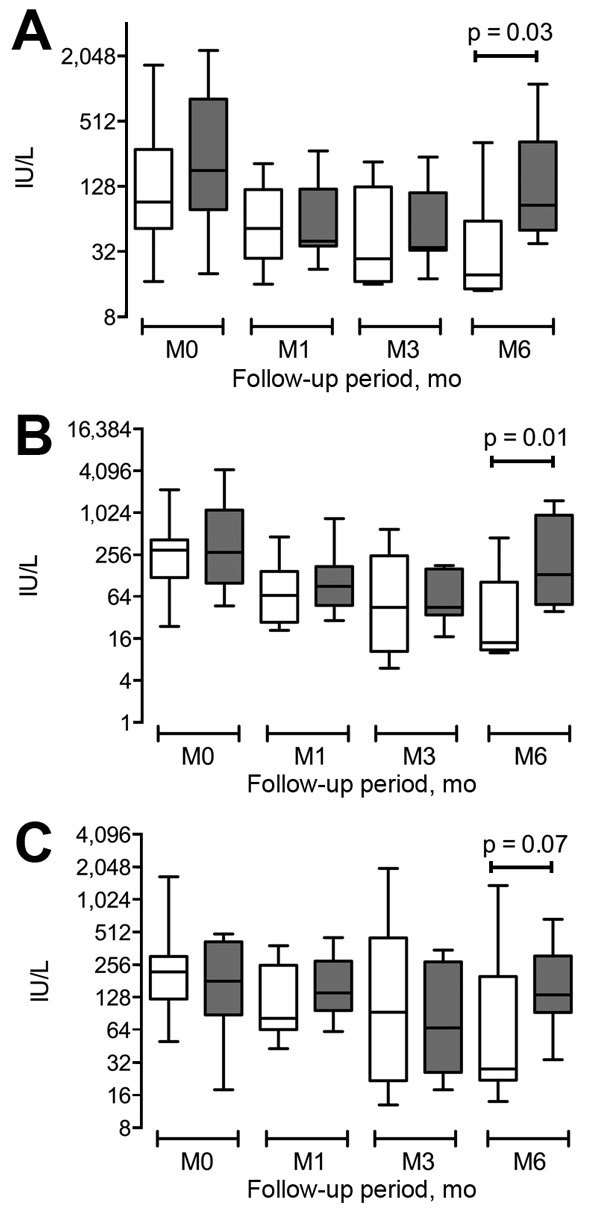
Liver test results for 26 patients at diagnosis of hepatitis E and at months 1, 3, and 6, Toulouse, France, 2003–2014. A) Aspartate aminotransferase ST; B) alanine aminotransferase; C) Gamma-glutamyl transferase.

The outcomes of both HEV infection and hematologic malignancies are summarized in Table 2. Twelve patients were treated with ribavirin after a median delay of 1 month (range 0.5–12 months) after the onset of hepatitis. Among the 14 patients with acute hepatitis E who did not receive ribavirin, 9 showed spontaneous clearance of HEV within the 2 months after diagnosis. The remaining 5 patients had prolonged HEV replication lasting >3 months, including 1 patient who had superimposed nonalcoholic steatohepatitis and had developed overt cirrhosis and refractory ascites. Immunosuppression withdrawal in 1 patient, who received allogeneic-SCT and in whom chronic hepatitis E developed, resulted in a severe transient incident of elevated liver enzymes, followed by spontaneous clearance of the virus. 

**Table 2:** Hepatitis E and hematologic malignancies outcomes of 26 patients observed at the University Hospital of Toulouse, France, 2003–2014*

Characteristic	Acute leukemia, n = 9	Indolent NHL, n = 8	Aggressive NHL, n = 3	Myeloma, n = 3	Others, n = 3
Chronic hepatitis	2 (22)	2 (25)	1 (33.5)	1 (33.5)	0
Ribavirin	3 (33.3)	4 (50)	2 (66.5)	1 (33.5)	2 (66.5)
HEV clearance	8 (89)	7 (87.5)	3 (100)	3 (100)	3 (100)
Postponed chemo/SCT	3 (33.3)	2 (25)	0	0	0
Hematologic complete response	5 (55.5)	7 (87.5)	2 (66.5)	0	1 (33.5)
Follow-up period, mo (range)	4.2 (0.9–51.7)	23.1 (5.6–105)	13.1 (3–29.1)	27 (12.8–83)	8.4 (1.5–10)
Deaths	4 (44.5)	1 (12.5)	1 (33.3)	1 (33.3)	1 (33.3)

*Values are no. (%) patients except as indicated. NHL, non-Hodgkin lymphoma; SCT, stem cell transplantation.

Oral ribavirin was administered to 12 patients at a median dose of 800 mg/d (range 600–1,000 mg/d); 1 patient had chronic hepatitis. In all patients, HEV RNA became undetectable after 30 (range 10–60) days, and liver blood tests were normalized after 2.5 (range 1–12) months. Ribavirin was given for a median time of 3 (range 0.5–10) months. The heterogeneity of timing and duration of treatment likely reflected the progressive introduction of ribavirin into current practice and the specific context of several patients. In general, 2 consecutive negative PCRs during 1 month were required to discontinue ribavirin. Three patients had abnormal liver test results and HEV recurred within the month after ribavirin treatment was withdrawn, including 2 who received ribavirin for 1 month only. Definitive virologic response was obtained after ribavirin retreatment. Thus, the combination of a 3-month intake of ribavirin, 2 negative HEV blood RNAs at month 2 and 3, and negative RNA levels in stools at month 3 seems to be an appropriate approach.

Overall, aspartate aminotransferase and alanine aminotransferase serum levels were significantly higher at 6 months in patients who did not receive ribavirin compared with patients who did (p = 0.03 and p = 0.01, respectively; Technical Appendix Table). Ribavirin was well tolerated among 10 of the 12 recipients and did not require specific red blood cells or erythropoietin support. One patient, 84 years of age, became anemic while receiving 1,000 mg of ribavarin per day, but treatment was continued. Treatment was discontinued for another patient, 41 years of age, when pruritus developed after 2 weeks of therapy.

## Conclusions

After SOT, chronic hepatitis develops in 60% of untreated HEV-infected patients (*3*). Here, we show that patients with hematologic malignancies are also at risk for chronic HEV infection. SOT patients showed a high response rate to ribavirin treatment (*11*). Because most hematologic malignancies required intensive immune treatments that should not be postponed, HEV infection could notably alter the therapeutic schedule of some patients (*5*,*6*). Cytotoxic treatments or SCT were postponed while awaiting HEV clearance for 5 patients, of whom 4 reached complete hematologic response. Among the 12 ribavirin-treated patients, 9 were able to complete the planned treatment that included SCT. Thus, although spontaneous but unpredictable HEV clearance could occur in some patients, therapeutic intervention is often mandatory to fully complete the therapeutic schedule of the underlying disease and to prevent chronic hepatitis. Confirming previous reports (*11*,*12*), a 3-month course of ribavirin induced complete and sustained HEV eradication in most if not all patients. Moreover, ribavirin may also decrease the risk for nosocomial HEV transmission among patients (*14*).

Our study has limitations. The retrospective nature prevented accurate assessment of the incidence of HEV infection among patients who have hematologic malignancies. Some obvious bias, including occurrence of HEV viremia without concomitant liver tests abnormalities or inability to ask patients about their exposure risk to HEV, also need further considerations.

By inducing a rapid clearance of HEV, ribavirin can prevent chronic hepatitis in patients with hematologic malignancies. Hepatitis E can induce chronic hepatitis and cirrhosis in patients with hematologic cancers; patients with liver enzyme abnormalities should be screened for HEV infection and a 3-month course of ribavirin proposed to avoid postponement in the treatment of patients who have the underlying disease as well as chronic hepatitis or cirrhosis.

Technical Appendix Characteristics and outcomes of patients with hematologic malignancies treated with ribavirin for prevention of chronic hepatitis.
