# Supplementary material for: Ribavirin for Chronic Hepatitis Prevention among Patients with Hematologic Malignancies
Source: Emerg Infect Dis. 2015 Aug;21(8):1466–9. doi: 10.3201/eid2108.150199 (PMC4517705; doi:10.3201/eid2108.150199)
Supplement: Technical Appendix — Characteristics and outcomes of patients with hematologic malignancies treated with ribavirin for prevention of chronic hepatitis. [file 15-0199-Techapp-s1.pdf]

Article DOI: http://dx.doi.org/10.3201/eid2108.150199

Ribavirin for Chronic Hepatitis Prevention among Patients with Hematologic Malignancies

Technical Appendix Table. Hematologic and virologic characteristics of patients with hematologic malignancies and outcome of administration of ribavirin for chronic hepatitis prevention*

Patient no.	Hematologic malignancy	Chemotherapy		Ribavirin (months)	Viremia (months)		Follow-up period (months)	Outcome	
		Before HEV	After HEV		Before Ribavirin	After Ribavirin		Malignancy	Hepatitis E
1	AML	Daunorubicin, cytarabin	Allo-SCT	No	0.5	NA	73	CR	Spontaneous clearance
2	AML (relapse)	Idarubicin, cytarabin, gemtuzumab, thiopurine, MTX	None	No	1	NA	3	Death (AML)	Spontaneous clearance
3	AML	Daunorubicin, cytarabin, allo-SCT conditioning	Allo-SCT	No	7	NA	52	CR	Spontaneous clearance
4	AML	Azacitidine	Azacitidine	No	3	NA	3	Death (AML)	Persistent viremia
5	B-ALL Phi +	Nilotinib, steroids, vincristine	Hyper-CVAD	Yes (10†)	1	1	11	Death (ALL)	CR
6	B-ALL Phi +	Nilotinib, Steroids, vincristine, cytarabin, MTX, auto-SCT, thiopurine	Nilotinib, thiopurine, MTX	No	1	NA	20	CR	Spontaneous clearance
7	AML	Azacitidine	Azacitidine	No	1	NA	4	Death (AML)	Spontaneous clearance
8	T-ALL	Daunorubicine, vincristine, asparaginase, steroids	None	Yes (3)	0.5	1	1.5	CR	CR
9	CML/B-ALL Phi +	Nilotinib, steroids, vincristine, allo-SCT	Steroids	Yes (3)	1	1	5	CR	CR
10	Mantle cell lymphoma	R-CHOP, R-DHAP	Allo/Auto-SCT	No	1	NA	105	CR	Spontaneous clearance
11	Anaplastic T-cell lymphoma	None	ACVBP and auto-SCT	No	9	NA	66	CR	Spontaneous clearance
12	Mantle cell lymphoma	R-CHVP, R-DHAP	Auto-SCT	No	1	NA	80	Relapse / CR	Spontaneous clearance
13	MDS and follicular lymphoma	Azacitidine	Azacitidine	No	6	NA	5.5	Death (cirrhosis)	Persistent viremia
14	Follicular lymphoma	R-CVP	R	Yes (7†)	0.5	1.5	12	CR	CR

Patient no.	Hematologic malignancy	Chemotherapy		Ribavirin (months)	Viremia (months)		Follow-up period (months)	Outcome	
		Before HEV	After HEV		Before Ribavirin	After Ribavirin		Malignancy	Hepatitis E
15	CLL	Allo-SCT, steroids		Yes (10)	0.5	1	7.5	CR	CR
16	CLL	RFC	Ofatuzumab, bendamustin	Yes (1)	0.5	0.5	24	CR	CR
17	CLL	R-Bendamustin	R-Bendamustin	Yes (3)	0.5	1	2	CR	CR
18	Burkitt's lymphoma	COPADEM, CYVE	Maintenance therapy, R-DHAOX	No	2	NA	13	Death (NHL)	Spontaneous clearance
19	DLBCL	R-ACVBP, R-DHAC, RICE	Auto-SCT, R	Yes (0.5)	7	2	29	CR	Spontaneous clearance
20	DLBCL	R-CHOP	R-CHOP	Yes (3)	1	1	3	CR	CR
21	Multiple myeloma	VAD, auto-SCT	Steroids, Pomalidomid	No	12	NA	71	PR	Spontaneous clearance
22	Multiple myeloma	Steroids, bendamustin, pomalidomide, C-PAD	None	Yes (5†)	1	1+2	13	PR	CR
23	Multiple myeloma	MP-Thal	None	No	1	NA	27	Death (AKI)	Spontaneous clearance
24	Hypereosinophilic syndrome	Imatinib, steroids	Nilotinib	No	0.5	NA	1.5	PR	Spontaneous clearance
25	Myeloproliferative neoplasm	Azacitidine	None	Yes	1.5	0.5	10	Death (AML)	CR
26	Granulocytic sarcoma	Daunorubicin, cytarabin	Flag-Idarubicin, Auto-SCT	Yes	0.5	0.5	8.5	CR	CR

*AML, acute myeloid leukemia; HEV, hepatitis E virus; Allo, allogenic; SCT, stem-cell transplant; NA, not applicable; CR, complete response; MTX, methotrexate; B-ALL, acute B-cell lymphoid leukemia; Phi, Philadephia chromosome; Hyper CVAD, fractionated cyclophosphamide, vincristine, adriamycin, dexamethasone; T-ALL, acute T-cell lymphoid leukemia; CML, chronic myeloid leukemia; R, Rituximab; CHOP, cyclophosphamide, doxorubicin, vincristine, prednisone; DHAP, dexamethasone, high dose aracytine, cisplatin, prednisone; Auto, autologous; ACVBP, doxorubicin, cyclophosphamide, vincristine, bleomycin, prednisone ; CHVP, cyclophosphamide, adriamycin, etoposide, prednisolone; MDS, myelodysplastic syndrome; CVP cyclophosphamide, vincristine, prednisone; CLL, chronic lymphoid leukemia; RFC, rituximab, fludarabine, cyclophosphamide; COPADEM, cyclophosphamide, vincristine, prednisolone, doxorubicine, hydrocortisone, methotrexate; CYVE, high dose cytarabine, etoposide; DHAOX, dexamethasone, oxaliplatin, cytarabine; DLBCL, diffuse large B-cell lymphoma; DHAC, dexamethasone, high dose aracytine, carboplatine, prednisone; ICE, ifosfamide, carboplatin, etoposide; PR, partial response; MP-Thal, melphalan, prednisone, thalidomide ; AKI, acute kidney injury.

†In these 3 patients, HEV relapse occurred within 1 month after the withdrawal of ribavirin and ribavirin was resumed; total time of ribavirin is given.
